# Paternal High-Protein Diet Programs Offspring Insulin Sensitivity in a Sex-Specific Manner

**DOI:** 10.3390/biom11050751

**Published:** 2021-05-18

**Authors:** Pengfei Gong, Danielle Bailbé, Lola Bianchi, Gaëlle Pommier, Junjun Liu, Stefania Tolu, Maria G. Stathopoulou, Bernard Portha, Valérie Grandjean, Jamileh Movassat

**Affiliations:** 1 Université de Paris, BFA, UMR 8251, CNRS, Team “Biologie et Pathologie du Pancréas Endocrine”, 75013 Paris, France; hbugpf@163.com (P.G.); danielle.bailbe@univ-paris-diderot.fr (D.B.); lolabanchi@orange.fr (L.B.); gaelle.pommier@paris7.jussieu.fr (G.P.); stefania.tolu@u-paris.fr (S.T.); bernard.portha@paris7.jussieu.fr (B.P.); 2 Shandong Institute of Endocrine and Metabolic Diseases, Shandong First Medical University, Jinan 250000, China; ftdxfish@yahoo.fr; 3 Université Côte d’Azur, Inserm, C3M, Team Control of Gene Expression (10), 06103 Nice, France; Maria.Stathopoulou@unice.fr (M.G.S.); Valerie.Grandjean@unice.fr (V.G.)

**Keywords:** paternal programming, high-protein diet, glucose homeostasis, endocrine pancreas, insulin sensitivity, insulin secretion, sperm small non-coding RNAs

## Abstract

The impact of maternal nutrition on offspring is well documented. However, the implication of pre-conceptional paternal nutrition on the metabolic health of the progeny remains underexplored. Here, we investigated the impact of paternal high-protein diet (HPD, 43.2% protein) consumption on the endocrine pancreas and the metabolic phenotype of offspring. Male Wistar rats were given HPD or standard diet (SD, 18.9% protein) for two months. The progenies (F1) were studied at fetal stage and in adulthood. Body weight, glycemia, glucose tolerance (GT), glucose-induced insulin secretion in vivo (GIIS) and whole-body insulin sensitivity were assessed in male and female F1 offspring. Insulin sensitivity, GT and GIIS were similar between F1 females from HPD (HPD/F1) and SD fathers (SD/F1). Conversely, male HPD/F1 exhibited increased insulin sensitivity (*p* < 0.05) and decreased GIIS (*p* < 0.05) compared to male SD/F1. The improvement of insulin sensitivity in HPD/F1 was sustained even after 2 months of high-fat feeding. In male HPD/F1, the β cell mass was preserved and the β cell plasticity, following metabolic challenge, was enhanced compared to SD/F1. In conclusion, we provide the first evidence of a sex-specific impact of paternal HPD on the insulin sensitivity and GIIS of their descendants, demonstrating that changes in paternal nutrition alter the metabolic status of their progeny in adulthood.

## 1. Introduction

It is now well established that environmental factors during perinatal life influence the health of individuals in adulthood. This is known under the concept of DOHaD (Developmental Origin of Health and Diseases) originally proposed by Barker and his collaborators [1,2]. While the vast majority of studies within the scope of early-life programming of adult-onset diseases deal with the influence of maternal environment, the past decade has witnessed an increasing interest towards the impact of the pre-conceptional paternal environment on the health of offspring [3]. Recently, increasing evidence has shown that the pre-conceptional nutritional status of fathers has profound impacts on the metabolic health of their progeny. This has been demonstrated through epidemiological data [4] and interventional studies in animals fed various diets such as high-fat diet [5,6], high-fat diet with additional treatment with the beta-toxic agent streptozotocin [7], or animals fed with a low-protein diet [8,9].

High-protein diets (HPD) and/or dietary protein supplements are becoming increasingly popular as a means to reduce obesity [10], to increase muscle mass or to improve physical performance [11,12]. Moreover, several studies suggested a beneficial effect of high-protein diets on insulin resistance and glucose homeostasis, independent of weight loss [13,14,15]. These observations have created a positive perception of high amounts of proteins as an essential factor for a healthy diet.

The influence of maternal high- or low-protein intake during gestation and/or lactation on offspring’s energy and glucose metabolism has been largely documented in humans and in various animal models. For instance, maternal low-protein diet was reported to affect hepatic and serum lipid content in offspring [16,17]. Low-protein during gestation and lactation predisposed to impaired glucose tolerance in male offspring as early as weaning [18], and impaired insulin sensitivity in adipose tissue and skeletal muscle in offspring at adult age [19]. Other studies have reported that low-protein diet during gestation resulted in a 50% decrease in the β cell mass in offspring [20,21]. Moreover, in both animal models and human populations, maternal intake of high-protein diet during pregnancy resulted in excessive development of the adipose tissue [22] and offspring’s overweight in adulthood [23]. However, to date, no study has assessed the impact of paternal consumption of high-protein diet/protein supplements on the health of their progeny.

As this novel nutritional habit develops in modern societies, especially among men at the age of reproduction, it is important to assess its impact on the metabolic phenotype of individuals, and, more importantly, to investigate its potential metabolic programming effects on the next generation(s). Since other nutritional interventions that are known to deteriorate glucose and energy homeostasis in F0 have repeatedly reported a negative impact on glucose homeostasis in their F1 progeny [5,6,7], we hypothesized that HPD feeding of fathers would also impact the metabolic phenotype of their offspring, potentially in a positive manner.

To test this hypothesis, we investigated the consequences of paternal high-protein diet ingestion on glucose and energy metabolism of their descendants. We demonstrated for the first time a sex-specific and bimodal impact of paternal HPD intake on insulin sensitivity and glucose-induced insulin secretion of their descendants (F1). This study provides yet another demonstration that changes in paternal nutrition have significant impact on the metabolism of their progeny in adulthood.

## 2. Materials and Methods

### 2.1. Animals

Male and female Wistar rats were obtained from our local colony maintained at Paris University animal core facility.

Experimental protocols were approved by Université de Paris Buffon ethical committee (no. CEB 10-2016 approved on 3 October 2016). Animal experiments have been carried out in accordance with France Directive no. 2013-118 in application of EU Directive 2010/63/EU.

### 2.2. Diets

The standard diet was SAFE D113, composed of 18.9% protein with about 22.5% of the energy provided by protein (SAFE, Villemoisson-sur-Ogre, France). The high-protein diet was composed of 43.2% protein, including 37% casein. In this diet, 48.6% of the energy was provided by protein (SAFE, Villemoisson-sur-Ogre, France). The standard diet contained 45% carbohydrate and 4.5% fat, while the high-protein diet contained 27.4% carbohydrate and 5.3% fat. The high-protein and the standard diets were isocaloric. The high-fat diet was SAFE 230 HF (SAFE, Villemoisson-sur-Ogre, France), and contained 36% fat.

### 2.3. Experimental Design

Rats were housed in a controlled environment (temperature 20 ± 1 °C, humidity 60%) with unlimited access to food and water and 12 h light/dark cycle. Fourteen male Wistar rats at the age of 10 weeks, weighing approximately 330 g, were housed (2 rats per cage) and divided into two groups. One group was fed with standard diet (SD group) (*n* = 6 rats). The other group was fed with a high-protein diet (HPD) for 2 to 3 months (HPD group) (*n* = 8 rats). Body weight, blood glucose, food and water consumption were monitored weekly. Blood glucose was tested on samples from the tail vein, by Accu-Check^®^ blood glucose test strips (Roche Diagnostics, Meylan, France) and Accu-Check^®^ Aviva glucometers (Roche Diagnostics, Meylan, France).

After 2 months feeding with high-protein (HPD) or standard diet (SD), the F0 rats (HPD and SD groups) were mated with 2 months old primiparous female Wistar rats fed with standard diet.

**Figure 1:**
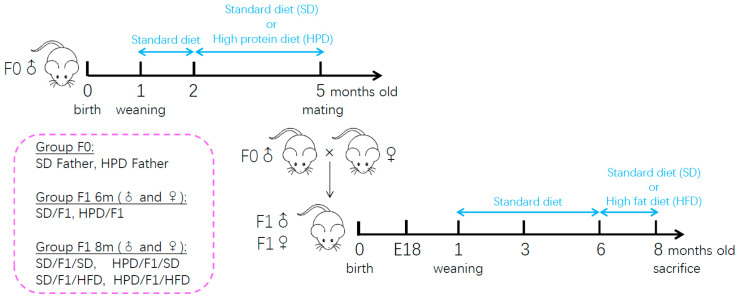
Schematic diagram of the experimental design.

The progenies (F1) were studied at the post-conception day 18 (E18) and in adulthood at the ages of 3, 6 and 8 months (Figure 1). For fetal studies, male and female fetuses from HPD fathers and SD fathers were recovered at the embryonic day 18 post-conception (E18). Fetuses from at least 4 different fathers were studied. Body weight of fetuses was measured. Pancreases were collected and weighed, then processed for histological analyses.

Both male and female offspring were studied. At birth, litters were restricted to 8 pups per dam. After weaning, the litters from HPD fathers were mixed randomly to minimize a possible litter effect, and fed with a standard diet until the age of 6 months. Distribution was conducted in such a manner that the experimental groups had the progenies of at least 4 different fathers. Male and female progenies were studied. The F1 rats from HPD father and SD father were named as HPD/F1 and SD/F1, respectively. At the age of 6 months, the F1 (male or female) from SD and HPD fathers were each divided in two groups, one fed with a high-fat diet (HFD) named SD/F1/HFD and HPD/F1/HFD, respectively. The other group of F1 from SD and HPD fathers was fed with standard diet for two months and named SD/F1/SD and HPD/F1/SD. Body weight, blood glucose, food and water consumption were monitored weekly from weaning to 6 months of age, and over the period of high-fat diet feeding. F1 male rats were euthanized at the age of 8 months (Figure 1). Liver and epididymal adipose tissue were dissected, then immediately frozen in liquid nitrogen and stored at −80 °C. Pancreas were dissected and processed for histological analyses.

### 2.4. Body Composition

Body composition was determined by a positron emission tomographic whole-body composition analyzer (EchoMRI, Houston, TX, USA).

### 2.5. Insulin Tolerance Test

After 6 h fasting with access to water, insulin tolerance tests (ITTs) were performed on non-anaesthetized Wistar rats at different ages. Human insulin was injected subcutaneously at the dose of 0.5 U/kg body weight. Samples of blood were collected at the tail vein before (0 min), and 15, 30, 60 and 90 min (or 120 min where indicated) after administration of insulin to measure glycemia. Blood glucose concentrations were determined using an Accu-Check^®^ Glucometer (Roche Diagnostics, Meylan, France).

### 2.6. Glucose Tolerance Test

After 6h fasting with access to water, intraperitoneal glucose tolerance tests (1 g glucose/kg body weight) were performed in non-anaesthetized Wistar rats at different ages. Blood was collected at the tail vein before (0 min), and 15, 30, 60 and 90 min after administration of glucose. Blood glucose concentration was determined using an Accu-Check^®^ Glucometer (Roche Diagnostics, Meylan, France). The plasma samples were stored at −20 °C for insulin determination with the enzyme-linked immunosorbent assay (ELISA) (ALPCO/Eurobio; Les Ulis, France).

### 2.7. Quantitative Real-Time PCR

Total RNA was extracted from rat liver and adipose tissue using the RNeasy mini Kit (Qiagen, Courtaboeuf, France). cDNAs of each RNA sample were synthesized with Maxima Reverse Transcriptase, using random hexamer primers. The primers were designed using OLIGO7 (the primer sets are described in Table 1). Quantitative real-time PCR amplification reactions were carried out in a Light Cycler 480 (Roche Applied Science, Meylan, France) using the SYBR Green I master kit (Roche, Meylan, France). All reactions were run in duplicate, with no template control. The PCR conditions were: 95 °C 10 min, followed by 45 cycles at 95 °C 10 s, 60 °C 10 s, and 72 °C 10 s. mRNA transcript levels of three housekeeping genes, *Tbp* (TATA-box-binding protein), *Cyclophilin A* and *Hprt1* (Hypoxanthine Phosphoribosyl transferase 1), were assayed. On the basis of the stability of the different genes, *Cyclophilin A* was retained for normalization of transcripts of rat liver and adipose tissue.

### 2.8. Histological Studies

Rats’ pancreases were fixed in aqueous Bouin solution for 24 h and embedded in paraffin (Labonord, Templemars, France). The entire pancreas was homogeneously sampled using a fixed-interval sampling method to avoid bias due to regional change in islets distribution, as previously described [24]. Briefly, each paraffin block was serially sectioned (5 μm) throughout its full-length, sections at a fixed interval were mounted on slides and stored at room temperature for future immune-staining.

Pancreatic sections were blocked for 1 h with 10% normal goat serum in Tris buffer saline and then probed for insulin using primary mouse anti-insulin antibody (Santa Cruz, France) (1:200). Sections were then incubated with an HRP conjugated anti-mouse secondary antibody (Jackson immunoResearch Laboratories, Ely, UK) (1:200). β cells areas were determined by morphometric analysis in pancreatic sections stained for insulin, using an OLYMPUS BX60 microscope equipped with Histolab 10.5.1 computer-assisted image analysis system software (Microvision Instrument, Evry, France). The relative β cell area in each stained section was calculated as the ratio of insulin positive cells area over the total pancreatic cells area [24,25]. At least six sections homogeneously distributed were analyzed for each pancreas.

### 2.9. Analysis of Islet Fibrosis

Pancreatic sections were stained with Picro Sirius Red Solution by 1 h incubation, followed by 20 s counterstaining with Papanicolaou’s solution. In each section, the total number of islets and the number of fibrotic islets (positive for Picro Sirius Red staining) were counted. Results were expressed as percentage of fibrotic islets over the total number of islets. At least 6 sections were analyzed for each pancreas.

### 2.10. Sperm Collection

Sperm from HPD and SD fathers were collected from the epididymis by squeezing. The cell suspension was centrifuged at 1000 rpm for 5 min, and the supernatant containing the spermatozoa was centrifuged at 3000 rpm for 15 min. To reduce contamination of somatic cells, the pellet was submitted to hypotonic shock by resuspension in water (250 μL), followed by the addition of 15 mL of PBS. The suspension was finally centrifuged at 3000 rpm for 15 min. The pellet was then stored at −80 °C for small RNA-sequencing analysis.

### 2.11. Small RNA-Sequencing Analysis

Total RNA was extracted by the TRIzol procedure (Invitrogen, Carlsbad, CA, USA). RNA was quantified in a Nanodrop ND-1000 spectrophotometer and RNA quality was checked using the Bioanalyzer-2100 equipment (Agilent Technologies, INC., Santa Clara, CA, USA). The experiment was carried out in triplicate. RNA libraries were prepared starting from 50 to 100 ng of total RNA from individual rats (*n* = 3 per group, 2 groups in total) and constructed using the Illumina TruSeq Stranded Small RNA Sequencing kit (Illumina, Evry, France) according to the manufacturer’s instructions. Sequencing was performed at the Genomix platform (Sophia-Antipolis, France) using the HiSeq 2500 (Illumina, Evry, France).

Read quality was assessed using FastQC and trimmed, against known common Illumina adapter/primer sequences, using trimmomatic. The SmallRNAs UCAGenomix pipeline with Illumina adaptor trimming was used, read sizes <15 nucleotides were discarded. In order to describe the general distribution of sperm small non-coding RNAs (sncRNAs), trimmed reads kept were mapped to small RNA databases using a recently developed annotation pipeline, SPORTS1.0 [26]. We used the default settings and database files for the rat genome Rnor_6.0, which are available on the Sports github (https//github.com/junchaoshi/sports1.0, accessed on 24 February 2021), while, for piRNAs, the piRNAdb v1.7.6 was used (www.pirnadb.org, accessed on 24 February 2021). Averages summarized over biotypes were based on the default annotation result output files. Differential expression analysis was performed on the SPORTS output files using DESeq2 R packages, following standard normalization procedures and the Benjamin and Hochberg method for multiple testing correction [27].

The baseMean for each gene, the mean of normalized counts of all samples, was at least 50 counts. NGS experiments have been deposited in the GEO Database with accession number GSE169157 online website accessed on 17 May 2021.

### 2.12. Data and Statistical Analysis

Results are expressed as means ± S.E.M. The statistical significance between means was assessed by Student’s *t* test when two groups were compared, or by ordinary one-way ANOVA followed by Fisher’s LSD test when more than two groups were compared. Two-way ANOVA followed by Sidak correction was used when comparing two parameters. The nonparametric multiple Mann–Whitney test with Holm–Sidak correction was used when data did not meet normal distribution. All analyses were performed using GraphPad Prism 9 (GraphPad Software, San Diego, CA, USA). *p* < 0.05 was considered statistically significant.

## 3. Results

### 3.1. Phenotype of Fathers

In this study, we used a diet containing 43.2% protein, mainly composed by casein, to mimic, at their best, the compositions of the protein supplements widely consumed by humans. In order to keep regularity regarding the duration of exposure, we decided to cover at least one full cycle of spermatogenesis (54 days in rat). Therefore, 10 weeks old male Wistar rats received the high-protein diet (HPD fathers) for 2 to 3 months, before being mated with Wistar females under standard diet (SD). Other male rats of the same age were fed with standard chow diet and used as controls (SD fathers).

**Figure 2:**
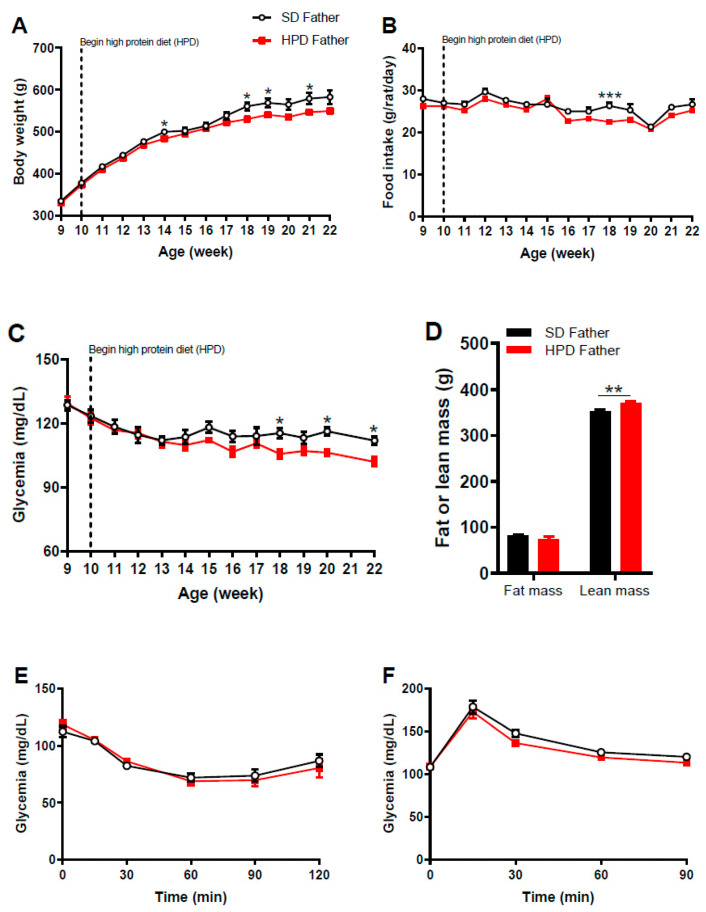

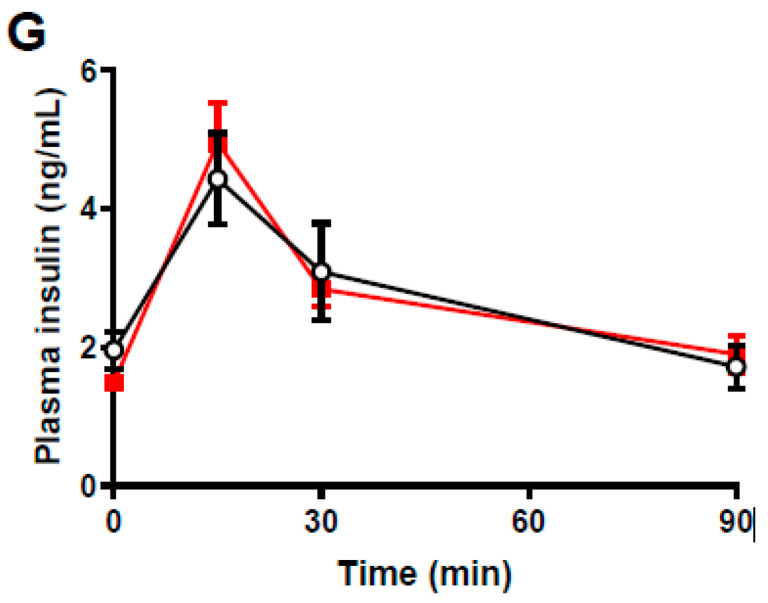
Evolution of body weight, food intake, blood glucose level, body composition and parameters of glucose homeostasis in HPD and SD fathers. Ten-week-old male Wistar rats were fed with high protein diet (HPD) or standard diet (SD) for up to 12 weeks (HPD Father, *n* = 8; SD Father, *n* = 6). (**A**) Body weight, (**B**) food intake and (**C**) glycemia were monitored weekly in HPD and SD rats. (**D**) Body composition, (**E**) insulin tolerance test (insulin, 0.5 U/kg body weight), (**F**) intraperitoneal glucose tolerance test (glucose, 1 g/kg body weight) and (**G**) measurement of glucose-induced insulin secretion (GIIS) were performed after 12 weeks of HPD feeding. Results are expressed as means ± SEM. * *p <* 0.05, ** *p* < 0.01 and *** *p* < 0.001.

First, we showed that the consumption of HPD resulted in reduced weight gain beginning at the age of 18 weeks post-natal in this group, as compared to the SD group (Figure 2A). This was associated with a decrease in food intake (Figure 2B). We also showed that from week 18 onward, blood glucose levels tended to decrease in the HPD group (Figure 2C). At the age of 22 weeks, the glycemia of HPD fathers decreased by 8.9% compared to SD fathers (102 ± 2.2 mg/dL vs. 112 ± 1.8 mg/dL, respectively, *p* < 0.05) (Figure 2C). However, the glycemic profiles during either ITT or IPGTT were similar between HPD and SD fathers (Figure 2E,F). There was no difference in glucose-induced insulin secretion during IPGTT between the two groups (Figure 2G). After 2-month treatment with high-protein diet, SD fathers and HPD fathers showed similar fat mass, but the lean mass was significantly increased in the HPD group compared to the SD group (370.9 ± 3.3 g vs. 353.2 ± 2.9 g, *p* < 0.01) (Figure 2D).

HPD and SD fathers were then mated with Wistar females under standard diet. Male and female offspring of both groups were studied either at the embryonic day 18 post-conception (E18), or in adulthood, at the ages of 3, 6 and 8 months.

### 3.2. Phenotype of F1 Fetuses

After mating, we did not observe any difference in the fertility of HPD fathers as compared to SD fathers. Because the conception, and subsequent growth and development, of fetuses are largely dependent on maternal physiology, we took specific caution to mate HPD and SD males with females of similar physiological conditions: all females were primiparous, were at the same age and had similar body weight.

We found that the number of fetuses per litter was not different between HPD and control groups (8 to 12 fetuses per dam). Moreover, the sex ratios among the litters were not statistically different in fetuses from the HPD and SD groups (55% female vs. 45% male).

**Figure 3:**
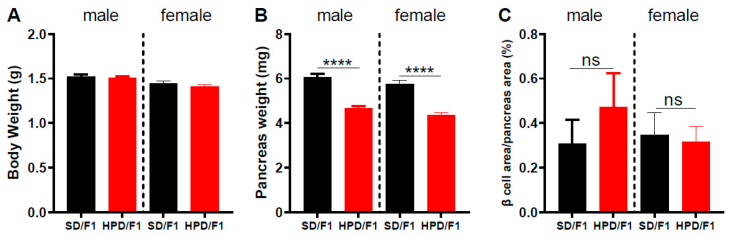
Body weight and pancreatic parameters of male and female fetal progenies from HPD and SD fathers at embryonic day 18 post-conception (E18). After 2 months of specific diet, HPD and SD fathers were mated with Wistar females fed with a standard diet. F1 male and female offspring were studied separately at the embryonic day 18 post-conception (E18). (**A**) Body weight, (**B**) pancreas weight and (**C**) the relative β cell area of male and female F1 fetuses at E18. SD/F1: offspring from SD father; HPD/F1: offspring from HPD father. Results are expressed as means ± SEM. *n* = 14–20 (**A**,**B**); *n* = 4 (**C**). **** *p* < 0.0001.

When the body weight of E18 fetuses was measured, we found that in both male and female fetuses from HPD fathers, the body weight was similar to those from SD fathers (Figure 3A). In contrast, a reduction in pancreas weight (*p* < 0.0001) was observed in both male and female fetuses from HPD fathers compared to fetuses from SD fathers (Figure 3B). There was no significant difference in β cell area between fetuses from the HPD group and the SD group, irrespective of their sex (Figure 3C).

### 3.3. Phenotype of Adult F1

The evolution of the body weight and glycemia were similar between age- and sex-matched F1 from HPD and SD fathers from weaning to 6 months of age (Figure S1). In order to assess the metabolic phenotype in offspring of HPD fathers, we measured glucose tolerance and insulin sensitivity through intraperitoneal glucose tolerance tests (IPGTT) and insulin tolerance tests (ITT), respectively.

**Figure 4:**
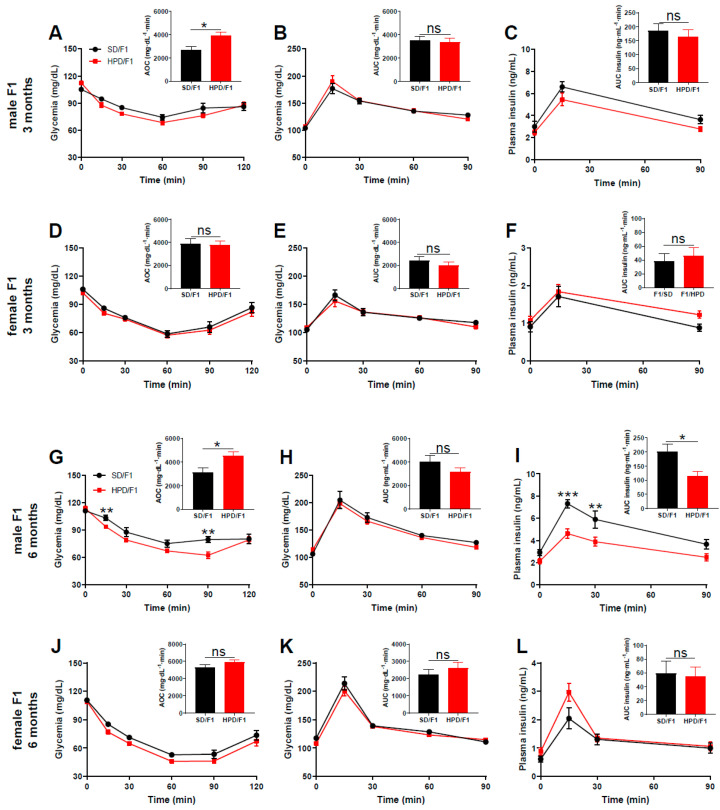
Parameters of glucose metabolism in 3 months old and 6 months old F1 from HPD and SD fathers. (**A**) Insulin tolerance test in male HPD/F1 and SD/F1 at the age of 3 months, (**B**) intraperitoneal glucose tolerance test in male HPD/F1 and SD/F1 at 3 months old and (**C**) glucose-induced insulin secretion in male F1 during IPGTT at the age of 3 months. (**D**) Insulin tolerance test in female HPD/F1 and SD/F1 at the age of 3 months, (**E**) intraperitoneal glucose tolerance test in female HPD/F1 and SD/F1 at the age of 3 months and (**F**) glucose-induced insulin secretion of female F1 during IPGTT at the age of 3 months. (**G**) Insulin tolerance test in male HPD/F1 and SD/F1 at the age of 6 months, (**H**) intraperitoneal glucose tolerance test in male HPD/F1 and SD/F1 at the age of 6 months and (**I**) glucose-induced insulin secretion in male F1 during IPGTT at the age of 6 months. (**J**) Insulin tolerance test in female HPD/F1 and SD/F1 at the age of 6 months, (**K**) intraperitoneal glucose tolerance test in female HPD/F1 and SD/F1 at the age of 6 months and (**L**) glucose-induced insulin secretion of female F1 during intraperitoneal glucose tolerance test at the age of 6 months. Area over the curve (AOC) (**A**,**D**,**G**,**J**) and area under the curve (AUC) (**B**,**C**,**E**,**F**,**H**,**I**,**K**,**L**) are shown in the insert. For all groups: Insulin tolerance test: insulin, 0.5 U/kg body weight; Intraperitoneal glucose tolerance test: glucose, 1 g/kg body weight. SD/F1: offspring from SD father; HPD/F1: offspring from HPD father. Seven to eleven animals were used in each group. Results are expressed as means ± SEM. * *p* < 0.05, ** *p* < 0.01 and *** *p* < 0.001.

At the age of 3 months post-natal, male offspring of HPD fathers (HPD/F1) exhibited enhanced insulin sensitivity (*p* < 0.05) compared to male F1 from SD fathers (SD/F1) (Figure 4A and Figure S2A). In contrast, there was no difference between the insulin sensitivity of female F1 from HPD and SD fathers (Figure 4D and Figure S2A).

Regarding the glucose tolerance at the age of 3 months, the glycemic profiles were identical in male HPD/F1 and SD/F1 (Figure 4B and Figure S2B), and no difference in glucose tolerance was apparent between F1 females from HPD and SD fathers (Figure 4E and Figure S2B). We also evaluated insulin secretion in response to glucose challenge in vivo. Interestingly, the glucose-induced insulin secretion was reduced slightly but not significantly in male HPD/F1 compared to male SD/F1 (Figure 4C and Figure S2C), while this parameter was unaffected in F1 females from HPD fathers when compared to F1 females from SD fathers (Figure 4F and Figure S2C).

At 6 months of age, insulin sensitivity was significantly enhanced in male HPD/F1 compared to SD/F1 (*p* < 0.05) (Figure 4G and Figure S2D), while the insulin sensitivity of HPD/F1 females was similar to that of SD/F1 females (Figure 4J and Figure S2D).

Regarding the glucose tolerance, no difference was seen in neither male nor female HPD/F1 when compared to the sex-matched SD/F1 (Figure 4H,K and Figure S2E). The glucose-induced insulin secretion (GIIS) measured during IPGTT revealed significantly reduced insulin secretion in 6 months old HPD/F1 males compared to SD/F1 males (*p* < 0.05) (Figure 4I and Figure S2F). At this age (6 months old), GIIS was similar between HPD/F1 and SD/F1 females (Figure 4L and Figure S2F).

### 3.4. Phenotype of Adult F1 under Metabolic Challenge

Next, in an additional set of experiments, we exposed male and female F1 from HPD and SD fathers to a high-fat diet for a period of two months and evaluated the impact of this metabolic challenge on the parameters of glucose homeostasis.

**Figure 5:**
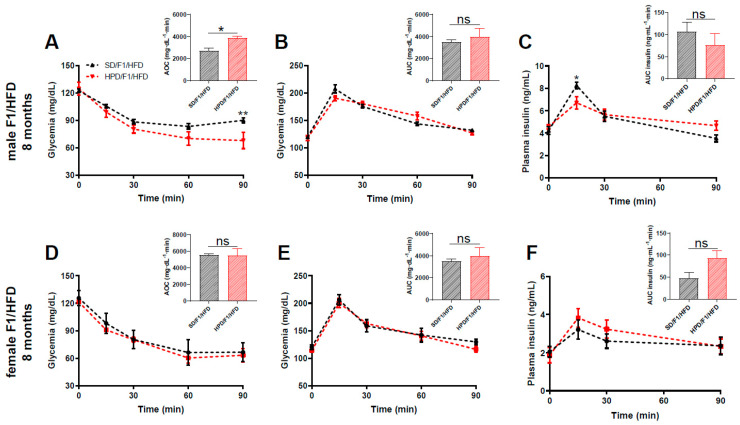
Parameters of glucose metabolism in 8 months old F1 from HPD and SD fathers following metabolic challenge. (**A**) Insulin tolerance test, (**B**) intraperitoneal glucose tolerance test and (**C**) glucose-induced insulin secretion in male HPD/F1/HFD and SD/F1/HFD, at the age of 8 months. (**D**) Insulin tolerance test, (**E**) intraperitoneal glucose tolerance test and (**F**) glucose-induced insulin secretion in female HPD/F1/HFD and SD/F1/HFD, at the age of 8 months. Area over the curve (AOC) (**A**,**D**) and area under the curve (AUC) (**B**,**C**,**E**,**F**) are shown in the insert. For all groups: Insulin tolerance test: insulin, 0.5 U/kg body weight; Intraperitoneal glucose tolerance test: glucose, 1 g/kg body weight. SD/F1/HFD: offspring from SD father fed with HFD; HPD/F1/HFD: offspring from HPD father fed with HFD. Four to six animals were analyzed in each experimental group. Results are expressed as means ± SEM. * *p* < 0.05 and ** *p* < 0.01.

As expected, the consumption of a high-fat diet (HFD) resulted in an increase in body weight. The body weight gain under HFD was similar between the SD/F1/HFD and HPD/F1/HFD groups in both sexes (Figure S3A,C). This was also observed in male SD and HPD offspring fed with a standard diet (Figure S3E,G). The evolution of non-fasted glycemia was similar between sex-matched progenies of SD fathers and HPD fathers, either under standard diet (Figure S3F,H) or high-fat diet (Figure S3B,D). Interestingly, when the insulin sensitivity was assessed in groups fed with a high-fat diet, we found that the F1 males from HPD fathers were less insulin resistant compared to those from SD fathers (*p* < 0.05) (Figure 5A), whereas HFD-fed females from HPD fathers were similar to HFD-fed females from SD fathers, regarding their whole-body insulin sensitivity (Figure 5D).

As expected, the ingestion of a high-fat diet was associated with glucose intolerance in both groups compared to the individuals, from respective fathers, fed with a standard diet (Figure S4). When HPD/F1/HFD were compared to SD/F1/HFD, the glucose tolerance profile was globally similar between the respective male and female groups (Figure 5B,E).

Intriguingly, when we compared the male SD/F1/SD rats to male HPD/F1/SD rats at the age of 8 months, a slight improvement of glucose tolerance was apparent in the latter group (Figure S5B), a feature which was not exhibited in this group at the age of 6 months (Figure 4H). At this time point, there was a slight, but not significant, improvement of insulin sensitivity in male and female HPD/F1/SD rats compared to sex-matched SD/F1/SD (Figure S5A,D). No difference was found in the glucose-induced insulin secretion in vivo between the two groups (Figure S5C,F).

The measurement of plasma insulin in blood samples collected during the IPGTT showed a slight reduction in insulin secretion, in response to glucose in vivo, in male HPD/F1/HFD rats compared to SD/F1/HFD rats (Figure 5C and Figure S2I), which was not observed in female HPD/F1/HFD (Figure 5F and Figure S2I).

### 3.5. Pancreatic Parameters

**Figure 6:**
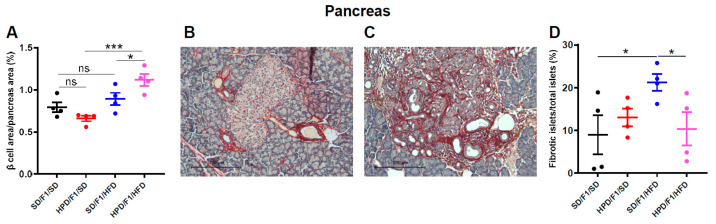

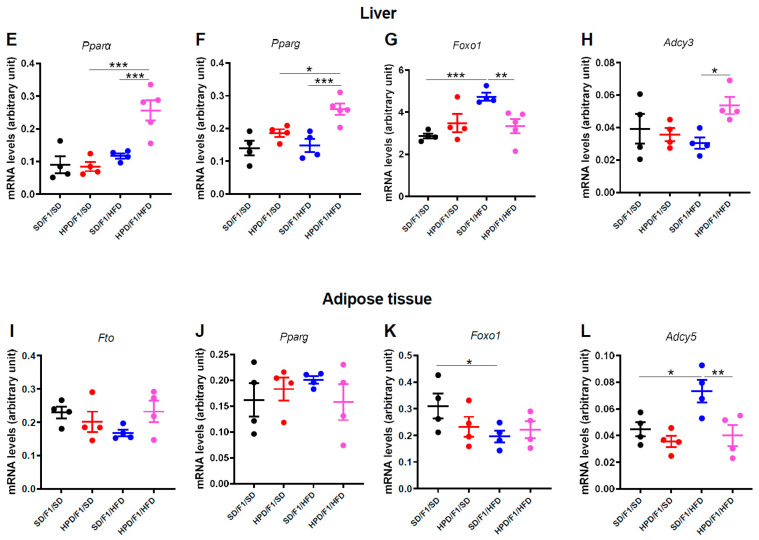
Pancreatic parameters and gene expression study in male offspring from SD and HPD fathers, before and after metabolic challenge. Morphometric analysis of β cells was performed on pancreatic sections of male SD/F1/SD, HPD/F1/SD, SD/F1/HFD and HPD/F1/HFD, at the age of 8 months. (**A**) The relative β cell area over total pancreas area was measured. (**B**) To detect islet fibrosis, pancreatic sections of 8 months old male SD/F1/HFD and (**C**) HPD/F1/HFD were stained with Picro Sirius Red solution, followed by counterstaining with Papanicolau’s solution. (**D**) Quantification of fibrosis expressed as the percentage of fibrotic islets over total number of islets per pancreas. Real-time PCR in male SD/F1/SD, HPD/F1/SD, SD/F1/HFD and HPD/F1/HFD at the age of 8 months was performed on samples from liver (**E**,**F**,**G**,**H**) and epididymal adipose tissue (**I**,**J**,**K**,**L**) for the indicated genes, and normalized to *Cyclophilin A*. The experiments were performed in duplicates. Four to five rats were analyzed in each experimental group. Results are expressed as means ± SEM. * *p* < 0.05, ** *p* < 0.01, *** *p* < 0.001.

After sacrifice, we collected the pancreases from male F1 for morphometric analyses. First, we showed that the relative β cell area over the total pancreatic area, representative of the β cell population within the pancreas, was not different between male HPD/F1 and SD/F1 under standard diet (Figure 6A).

After 2 months of high-fat diet, as described in the literature [28], signs of disrupted islet structure, and notably, islet fibrosis were apparent in both HPD/F1/HFD and SD/F1/HFD groups (Figure 6B,C). However, when the percentage of fibrotic islets over the total number of islets was evaluated in the two groups, we found a significantly lower percentage of fibrotic islets in the HPD/F1/HFD group compared to the SD/F1/HFD group (*p* < 0.05) (Figure 6D), suggestive of a rather protective effect of paternal high-protein diet on the pancreas of their male progeny.

The expansion of the β cell mass called “plasticity” has been described in response to increased insulin demands in conditions such as insulin resistance induced by HFD [28]. In our study, we showed a non-significant increase in the relative β cell area in the SD/F1/HFD group compared to the SD/F1/SD group, while a significant increase in the β cell area was observed in the HPD/F1/HFD rats compared to the HPD/F1/SD rats (*p* < 0.05) (Figure 6A), reflecting a preserved β cell plasticity in these individuals.

### 3.6. Gene Expression Studies

In order to further explore the metabolic changes observed in males F1 from HPD fathers, we investigated the expression of a number of genes in the liver and adipose tissue. The results are reported in Figure 6E–L. It should be noted that the observed differences were mainly between HPD/F1/HFD and the other groups.

In the liver, we showed that the expression of *Pparα*, *Pparg* and *Adcy3* was upregulated in the HPD/F1/HFD group compared to the other groups. Moreover, *Foxo1* expression was upregulated in the SD/F1/HFD group compared to the HPD/F1/HFD and the SD/F1/SD group.

In contrast, the HFD-induced upregulation of *Adcy5* observed in the adipose tissue of SD/F1/HFD rats was lacking in the HPD/F1/HFD group. Collectively, changes in the above mediators of insulin sensitivity/insulin resistance in the HPD/F1/HFD rats could contribute to the enhanced insulin sensitivity exhibited by these animals in the context of metabolic challenge.

### 3.7. Spermatic Small RNA-Sequencing Analysis

**Figure 7:**
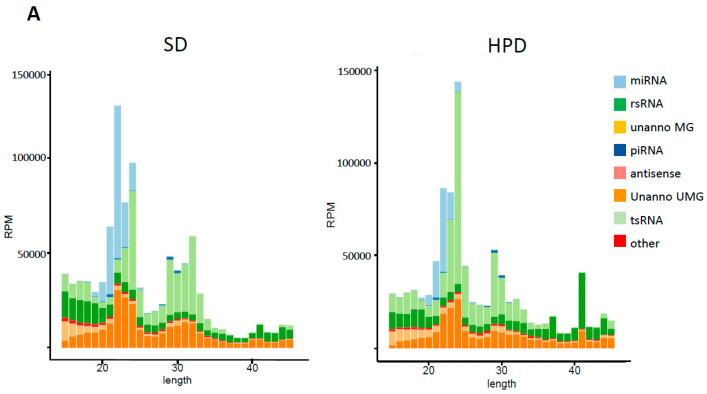

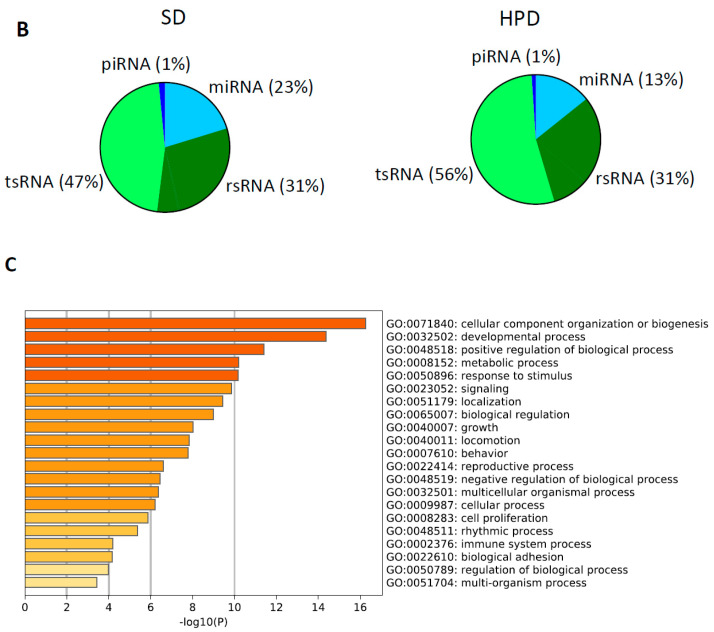
Deep sequencing analysis of small RNAs from SD and HPD spermatozoa. (**A**) Length distribution of small RNAs from SD and HPD spermatozoa. (**B**) Mean proportion of each small RNA population across each group (n = 3). (**C**) Enrichment of biological categories with 2464 gene targets of deregulated miRNAs in the HPD spermatozoa.

Thus, paternal HPD-feeding affects metabolic parameters, namely insulin sensitivity, of the progenies, suggesting that sperm epigenome is sensitive to the HPD. Spermatic small non-coding RNAs (sncRNAs) are known vectors of epigenetic intergenerational inheritance of metabolic pathologies, as revealed by studies with nutritional interventions such as paternal HFD-, LPD- and Western Diet feeding [28,29,30,31,32]. Here, we determined whether spermatic sncRNAs might be potential vectors of the physiological modifications observed in the progenies from HPD fathers, by analyzing the spermatic small non-coding RNA signature of the HPD fathers. As shown in Figure 7, distribution analysis of sncRNA populations revealed a marked decrease in miRNA population and an increase in tRNA-derived fragments in the HPD groups. On the other hand, length distribution analysis of ribosomal-derived small RNA (rsRNA) evidenced a specific HPD rsRNA signature with a 42 nt peak corresponding to mt-rRNA fragments whose biological significance remains unclear.

Focusing on the individual piRNA, tsRNA and miRNA differentially expressed between SD and HPD fathers (fold change ≥ 2 or ≤−2, adjusted *p* ≤ 0.05 and meanBase > 200), we identified 2 upregulated piRNAs, 46 deregulated tsRNA fragments (25 downregulated and 21 upregulated) and 56 deregulated miRNA (55 down and 1 up) (Supplementary Materials Table S1). Interestingly, 21 out of 56 deregulated miRNAs are found to be biologically involved in insulin sensitivity (Supplementary Materials Table S2). Furthermore, using the miRDP database, we identified 2464 gene targets of these 56 deregulated miRNAs (prediction score ≥ 80) [33]. Metascape analysis [34] performed with all of these gene targets showed high enrichment, with high statistical significance (−log_10_(*p*) range of 10–16), of developmental processes and regulation of biological processes (positive regulation of the metabolic process and biological process) (Figure 7C).

## 4. Discussion

This study aimed at the evaluation of the impact of paternal high-protein diet on the metabolic health of the descendants. High-protein diets have been promoted on the basis of their role in body’s energy homeostasis and they have been proposed as an efficient means for weight loss in overweight or obese individuals [10,35]. Several studies have also reported an improved glucose homeostasis, potentially by direct effects on insulin resistance and insulin secretion [13,14,15]. However, depending on the duration of the consumption of high-protein diets and the source of dietary proteins, the reported effects largely vary and some studies even show detrimental effects of the consumption of excess protein on glucose homeostasis [36,37,38,39]. Importantly, negative effects on general health have also been reported. For instance, long term excessive protein intake may be detrimental to individuals with a history of kidney dysfunction [40]. With the popularization of protein consumption above the recommended dietary allowance (0.8 g/kg body weight/day for adults), especially among young men in age of reproduction, it is important to investigate the effects of this type of diet, not only on the metabolic health of the individuals exposed to it, but also on their progeny. In our study, we used a high-protein diet, which was enriched in casein, to keep analogy with the most widely consumed protein supplements, which are casein-rich dietary proteins. We showed that up to 3 months of direct exposure to HPD produced beneficial metabolic effects on fathers (F0), as reflected by decreased body weight gain, increased lean mass, and also reduced blood glucose levels. It should be noted that, in this study, we used an isocaloric diet, in which high-protein content was associated with a low proportion of carbohydrates. This could be, in part, accountable for the lower blood glucose levels in HPD-fed males compared to that exhibited by males fed with SD.

In addition to the effects of HPD in adult male rats, the main objective of our study was to determine its effects on the next generation (F1). First, we found that paternal HPD did not significantly alter the size of litters, nor did it influence the sex ratio within the litters at E18 (55% females–45% males) when compared to litters from SD fathers. In contrast to our findings, sex ratio modification in favor of females was reported when fathers were fed with a low-protein diet [41].

The assessment of the adult descendants of HPD fathers revealed an alteration of offspring’s metabolic parameters in a sex-specific manner, affecting only the male progeny, while the metabolic traits of female F1 remained unaltered compared to age-matched female offspring of SD fathers. In studies on early-life paternal or maternal programming of diseases, wherever F1 from both sexes were investigated, different phenotypes among male and female offspring are often reported. The reasons for this sexual dimorphism are certainly multiple, but the role of estrogens might be a likely mechanism, as estrogens have been shown to exert protective actions against cardiovascular diseases and type 2 diabetes [42,43].

To the best of our knowledge, to date, there are no other reports on the consequences of high-protein diet intake by fathers, on the metabolic health of their progeny. In contrast, the effects of maternal consumption of either high-protein or low-protein diets during gestation and/or lactation have been largely documented. Rat fetuses exposed to maternal protein restriction during the third part of pregnancy have a dramatic decrease in their β cell mass [44]. Moreover, high-protein diet during gestation, but not lactation, resulted in increased susceptibility to obesity and to glucose intolerance in female offspring [22].

During the last decade, studies based on other types of nutritional interventions in fathers have shown profound effects on the metabolic phenotype of their offspring. For instance, in mice, consumption of a high-fat diet by male genitors induced glucose intolerance and β cell dysfunctions exclusively in female offspring [5,6,45]. In addition, lifestyle interventions such as paternal exercise have been shown to have protective effects on the metabolic health of offspring, and decrease the risk of T2D induced by a high-fat diet [46]. Recently, Watkins et al. have investigated the impact of a low-protein diet ingested by fathers on the cardiovascular and metabolic function of progeny in a mouse model [41]. They report vascular dysfunction and glucose intolerance in both male and female offspring, while only adult male offspring developed hypotension and elevated heart rate [41].

Our present study suggests a beneficial effect of HPD consumption by fathers, on their male progeny, in terms of insulin sensitivity. Indeed, as early as at 3 months of age, increased whole-body insulin sensitivity was apparent in male F1 from HPD fathers, which was sustained until the age of 6 months. Intriguingly, paternal HPD intake resulted in reduced in vivo insulin secretion, in response to glucose, in HPD/F1 compared to SD/F1. A plausible explanation for the reduced insulin secretion in the male offspring of HPD fathers would be that the decreased insulin secretion might be an adaptive response. Indeed, one could speculate that in our model, the insulin response to intraperitoneal glucose during IPGTT is appropriately reduced to accommodate the greater whole-body insulin sensitivity. This feature has been reported in a study in cholecystokinin-deficient mice, which exhibited adaptive reduced insulin secretion in the face of increased insulin sensitivity [47]. However, another interpretation for these results is that the β cell secretory function could be negatively affected by paternal HPD consumption. The effects of HPD consumption by fathers on the pancreatic phenotype of the progeny has not been investigated elsewhere. Only one study has investigated the effects of taurine supplementation in obese fathers, and showed that paternal intake of this specific amino acid partially reversed the deleterious effects of paternal obesity on insulin secretion and islet morphology in F1 offspring [48]. Interestingly, taurine has also been implicated in normal fetal β cell function during development [49].

Thus, the above studies point to a rather positive effect of paternal supplemental amino-acid intake towards β cell function in their progeny, which is in contrast with the reduced insulin secretion found in the male progeny of HPD fathers in our study. It is established that reduced glucose-induced insulin secretion in vivo could be caused by a decreased number of pancreatic β cells and/or a deficiency in the secretory capacity of these cells. Our morphometric analysis in male HPD/F1 showed that the β cell mass and islet morphology were unaltered in the adult male offspring of these rats compared to their SD/F1 counterparts. Moreover, the development of β cells during the fetal life (E18 days) was similar in fetuses from HPD fathers and those from SD fathers. Further, the expression of key transcription factors involved in pancreas development was not affected in the pancreases of fetuses from HPD fathers (data not shown). A high-fat diet is known to induce islets fibrosis in rodents [28]. Interestingly, when fed with a high-fat diet for two months, male offspring of HPD fathers (HPD/F1/HFD group) exhibited a smaller percentage of fibrotic islets compared to the islets of SD/F1/HFD males, suggestive of a rather protective effect of paternal HPD. Moreover, the so-called plasticity of β cells, illustrated by the increase in the β cell mass in the face of insulin resistance, was preserved and even increased in the male progeny of HPD fathers fed with a high-fat diet for 2 months. Collectively, these data indicate that the reduced glucose-induced insulin secretion in vivo in F1 from HPD fathers is not caused by the reduction in β cell number, nor their secretory capacity, but rather is an adaptive mechanism in response to increased insulin sensitivity. Nevertheless, a potential detrimental effect of paternal high-protein intake in specific contexts, such as congenital or acquired impairment of insulin secretion in susceptible individuals, should not be overlooked.

Our additional experiments with high-fat diet during two months in F1 from HPD and SD fathers revealed that HPD/F1/HFD males were less insulin resistant than animals from SD fathers (SD/F1/HFD), suggesting that the improvement of insulin sensitivity is sustained even under a metabolic challenge.

Gene expression studies revealed that a number of genes involved in the regulation of insulin sensitivity were modulated in HPD/F1 males exposed to HFD. *Pparα* and *Pparg* are important integrators of cellular glucose and lipid metabolism and a positive regulator of insulin sensitivity in the liver [50]. In our study, we found that the expression of *Pparg* was upregulated in the liver of HPD/F1/HFD. The expression of *Adcy3*, the gene encoding for adenylate cyclase 3, was also higher in the liver of HPD/F1/HFD males compared to SD/F1/HFD. *Adcy3* has been implicated in the regulation of body adiposity [51,52], but also has metabolic roles in the liver and muscle [53,54]. Interestingly, reduced expression of hepatic *Adcy3* results in increased vulnerability to diet-induced hepatic insulin resistance in mice [55]. Therefore, we could speculate that increased expression of this gene in HPD/F1/HFD rats could contribute to the enhanced insulin sensitivity found in this group compared to the SD/F1/HFD group. The expression of another Adenylate cyclase, namely the *Adcy5*, was modulated in the adipose tissue of HPD/F1/HFD rats. Anja et al. have reported that changes in *Adcy5* expression were related to adverse fat distribution and adipose tissue dysfunction, and that mice fed with a high-fat diet have increased expression of *Adcy5* mRNA [56]. In line with this report, here we show that SD/F1 fed with HFD have higher levels of *Adcy5* mRNA in the adipose tissue when compared to control SD/F1 on a regular diet, whereas HPD/F1 males did not increase their expression of *Adcy5* in the epididymal adipose tissue following the metabolic challenge (HPD/F1/HFD group), suggesting a programmed protective effect of paternal HPD intake on this parameter.

We have also found differences in the hepatic expression of *Foxo1*. *Foxo1* is an essential factor in the regulation of energy and glucose metabolism [57]. Abnormal expression of *Foxo1* protein is associated with metabolic disorders [58] and the use of *Foxo1* inhibitors resulted in the improvement of glucose metabolism [59]. In our study, the ingestion of the high-fat diet resulted in the upregulation of *Foxo1* in the liver of SD/F1/HFD compared to SD/F1/SD rats but not in HPD/F1/HFD rats. This could contribute to the improved insulin sensitivity observed in HPD/F1/HFD.

Overall, the results of our gene expression study point to the upregulation of genes positively involved in insulin sensitivity, and the downregulation of genes implicated in insulin resistance, in the HPD offspring exposed to a metabolic challenge.

The enhanced insulin sensitivity reported in our study could be associated with epigenetic paternal germline modifications induced by paternal HPD exposure. Based on several studies showing that changes in small non-coding RNA occur in relation to the nutritional environment of paternal sperm and could act as vectors of epigenetic intergenerational inheritance of diet-induced epigenetic modifications [29,30,31,32], we deeply analyzed the spermatic small non-coding RNA signature of the HPD fathers.

We identified a specific spermatic small non-coding RNA signature in HPD fathers, with a significant number of deregulated miRNA compared to SD fathers. Interestingly, 21 out of 56 deregulated miRNA were involved in insulin sensitivity, as indicated in Supplementary Materials (Table S2). To just cite a few, we found that miR378a was downregulated in the sperm of HPD fathers compared to SD fathers. Interestingly, overexpression of miR-378 has been shown to induce hepatic insulin resistance, suggesting a negative metabolic role for this miRNA [60]. In addition, we found a downregulation of miR-199a-3p in the sperm of HPD fathers. miR-199a-3p has been reported for its role in the modulation of obesity-associated insulin resistance and inflammatory responses [61]. This miRNA is upregulated in the plasma of T2D patients [62]. These data were consistent with a potential role of the small RNA of sperm as a vector of epigenetic inheritance in our model.

To conclude, in this study, we have added to the growing body of evidence linking the pre-conception nutritional status of fathers to the metabolic status of their progeny in adulthood. Our findings, which reveal that paternal high-protein diet enhances insulin sensitivity in male offspring, constitute a novel and potentially important observation and warrant additional investigation to uncover the associated molecular mechanisms. We have also established that offspring of HPD fathers have decreased GIIS. Although it is most likely that this is an adaptive mechanism in response to increased insulin sensitivity, a potential detrimental effect of paternal high-protein diet intake in the context of congenital impairment of insulin secretion should not be overlooked, and warrants further attention. As the consumption of protein-based meals and protein powders as nutritional supplements are becoming popular among young men, investigations of their impact on the metabolic health of their descendants should be extended.

## Figures and Tables

**Table 1:** List of rat primers used in this study.

Gene		Sequence
*Foxo1*	F	CTCACACATCTGCCATGAACCG
	R	AAATCCAAGGTATCTCCGTCCA
*Pparg*	F	CCACACTATGAAGACATCCCGTT
	R	ATGCTTTATCCCCACAGACTCG
*Pparα*	F	CTTCATCACCCGAGAGTTCCT
	R	TCATCCAGTTCGAGGGCATT
*Adcy3*	F	CGGCCATGGTGGAGATACTTA
	R	CCAAGATGAGAAGGCCACGA
*Adcy5*	F	GGGAGAACCAGCAACAGG
	R	CATCTCCATGGCAACATGAC
*Fto*	F	CGCCGCATGTCAGACCTTC
	R	TCCACTTCATCATCGCAGGAC
*Cyclophilin A*	F	AACCCCACCGTGTTCTTCGAC
	R	TGCCTTCTTTCACCTTCCCAA
*Hprt1*	F	TTGTTGGATATGCCCTTGACT
	R	CCGCTGTCTTTTAGGCTTTG
*Tbp*	F	CGTGAATCTTGGCTGTAAACTTGA
	R	GCTGCTAGTCTGGATTGTTCTTCA

## Data Availability

The data presented in this study are available on request from the corresponding author.

## Supplementary Materials

The following are available online at https://www.mdpi.com/article/10.3390/biom11050751/s1, Figure S1: Evolution of body weight and glycemia of male and female F1 from HPD and SD fathers from weaning to 24 weeks of age, Figure S2: Comparative of insulin tolerance test (ITT) and intraperitoneal glucose tolerance test (IPGTT) between male F1 and female F1, Figure S3: Evolution of body weight evolution and glycemia in male and female offspring fed with high-fat diet, Figure S4: Insulin tolerance test and intraperitoneal glucose tolerance test in 8 months old F1 from HPD and SD fathers fed with standard diet or high-fat diet, Figure S5: Parameters of glucose homeostasis in 8 months old F1 from HPD and SD fathers fed with standard diet, Table S1: Small RNA content of sperm from HPD and SD fathers, Table S2: deregulated miRNA involved in insulin sensitivity.
