# Supplementary material for: Paternal High-Protein Diet Programs Offspring Insulin Sensitivity in a Sex-Specific Manner
Source: Biomolecules. 2021 May 18;11(5):751. doi: 10.3390/biom11050751 (PMC8157381; doi:10.3390/biom11050751)
Supplement: Supplementary file 1 [file biomolecules-11-00751-s001.zip › Supplementary Figures.pdf]

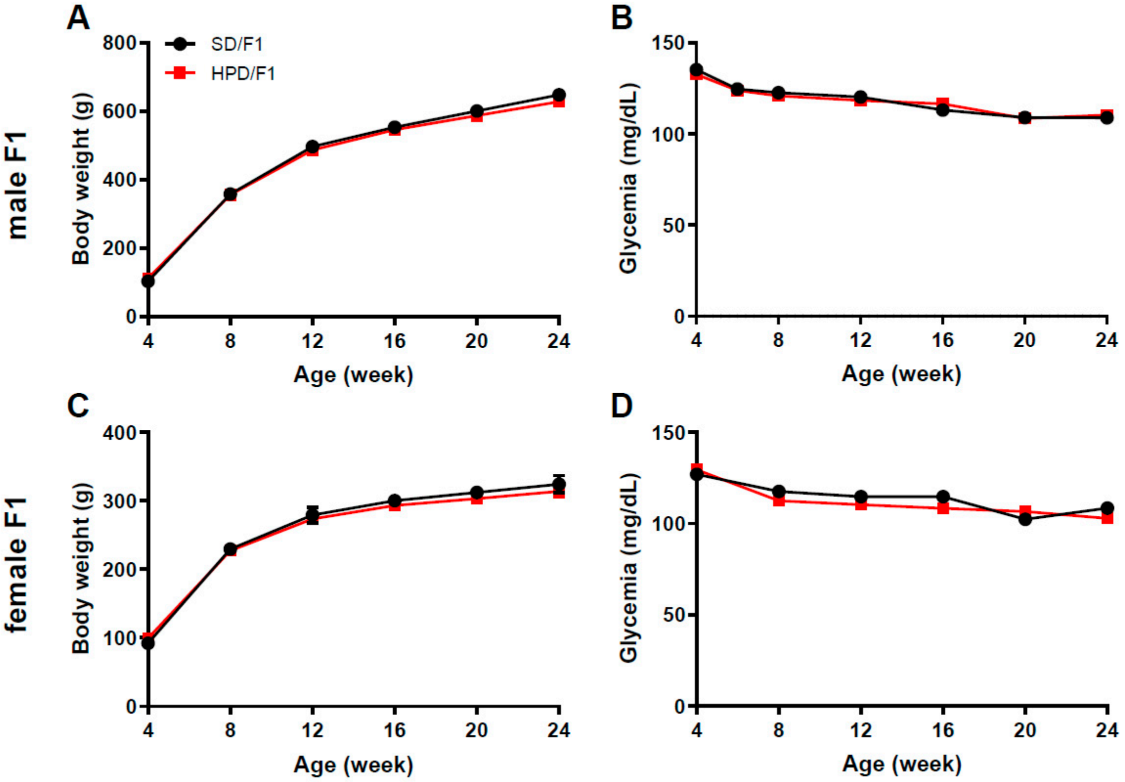

Figure S1. Evolution of body weight and glycemia of male and female F1 from HPD and SD fathers from weaning to 24 weeks of age. (**A**) Body weight, (**B**) glycemia of male F1, (**C**) body weight, (**D**) glycemia of female F1 from SD and HPD fathers were monitored monthly. SD/F1: offspring from SD father; HPD/F1: offspring from HPD father. n= 15-21 (A, C); n= 7-10 (B, D).

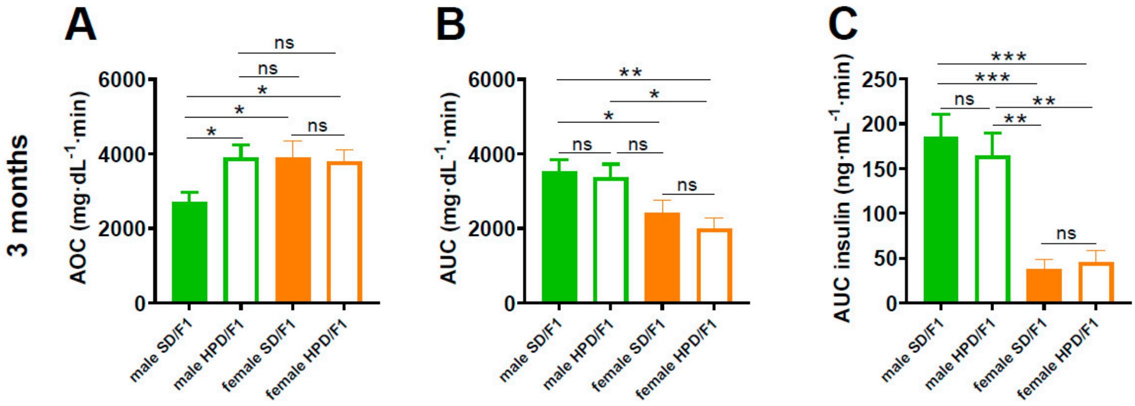

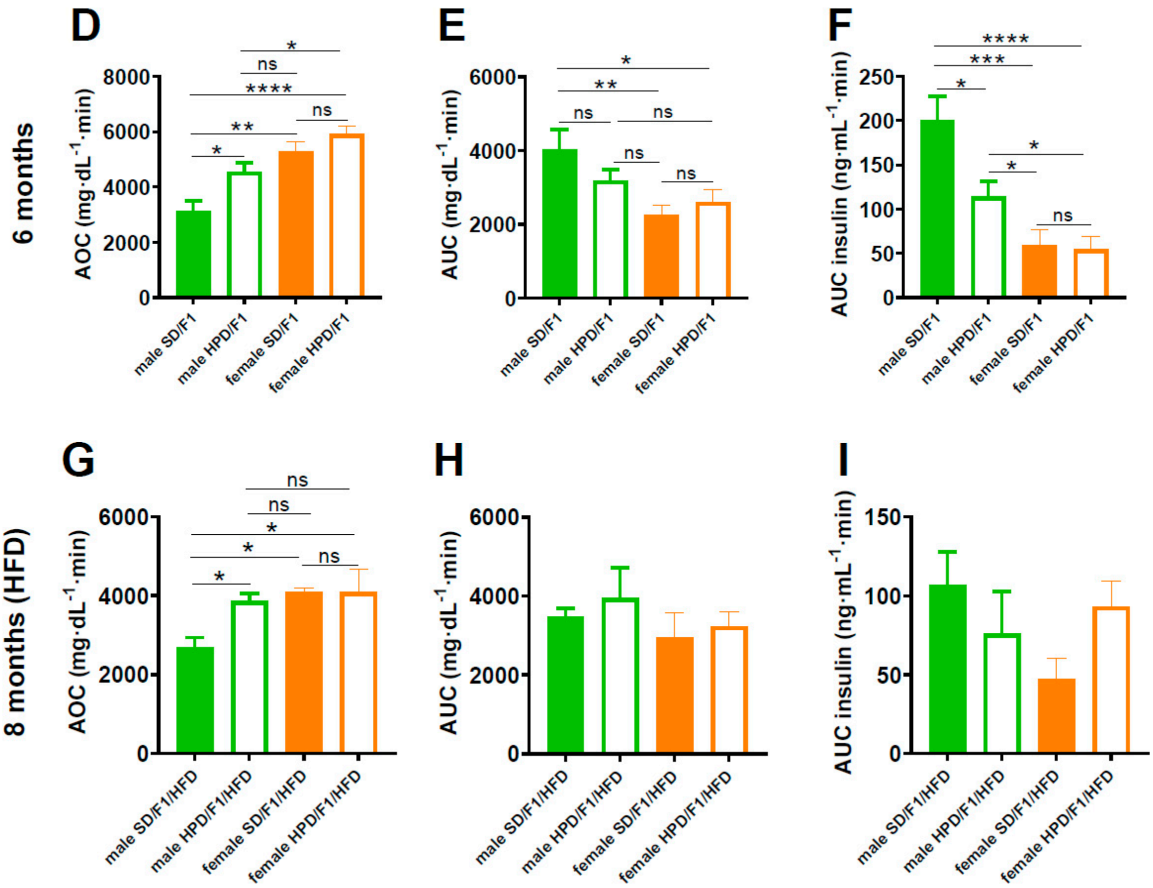

Figure S2. Comparative of insulin tolerance test (ITT) and intraperitoneal glucose tolerance test (IPGTT) between male F1 and female F1. Comparison of (**A**) Area over curve (AOC) of ITT, (**B**) area under the curve (AUC) of IPGTT and (**C**) AUC of glucose-induced insulin secretion during IPGTT among male SD/F1, male HPD/F1, female SD/F1 and female HPD/F1, at the age of 3 months. Comparison of (**D**) AOC of ITT, (**E**) AUC of IPGTT and (F) AUC of glucose-induced insulin secretion during IPGTT among male SD/F1, male HPD/F1, female SD/F1 and female HPD/F1, at the age of 6 months. Comparison of (**G**) AOC of ITT, (**H**) AUC of IPGTT and (**I**) AUC of glucose-induced insulin secretion during IPGTT among male SD/F1/HFD, male HPD/F1/HFD, female SD/F1/HFD and female HPD/F1/HFD, at the age of 8 months. SD/F1: offspring from SD fathers; HPD/F1: offspring from HPD fathers; SD/F1/HFD: offspring from SD father fed with HFD; HPD/F1/HFD: offspring from HPD father fed with HFD. An ordinary one-way ANOVA followed by Fisher's LSD test was used for the comparisons. Results are expressed as means ± SEM. *p<0.05, **p<0.01, ***p<0.001 and ****p<0.0001.

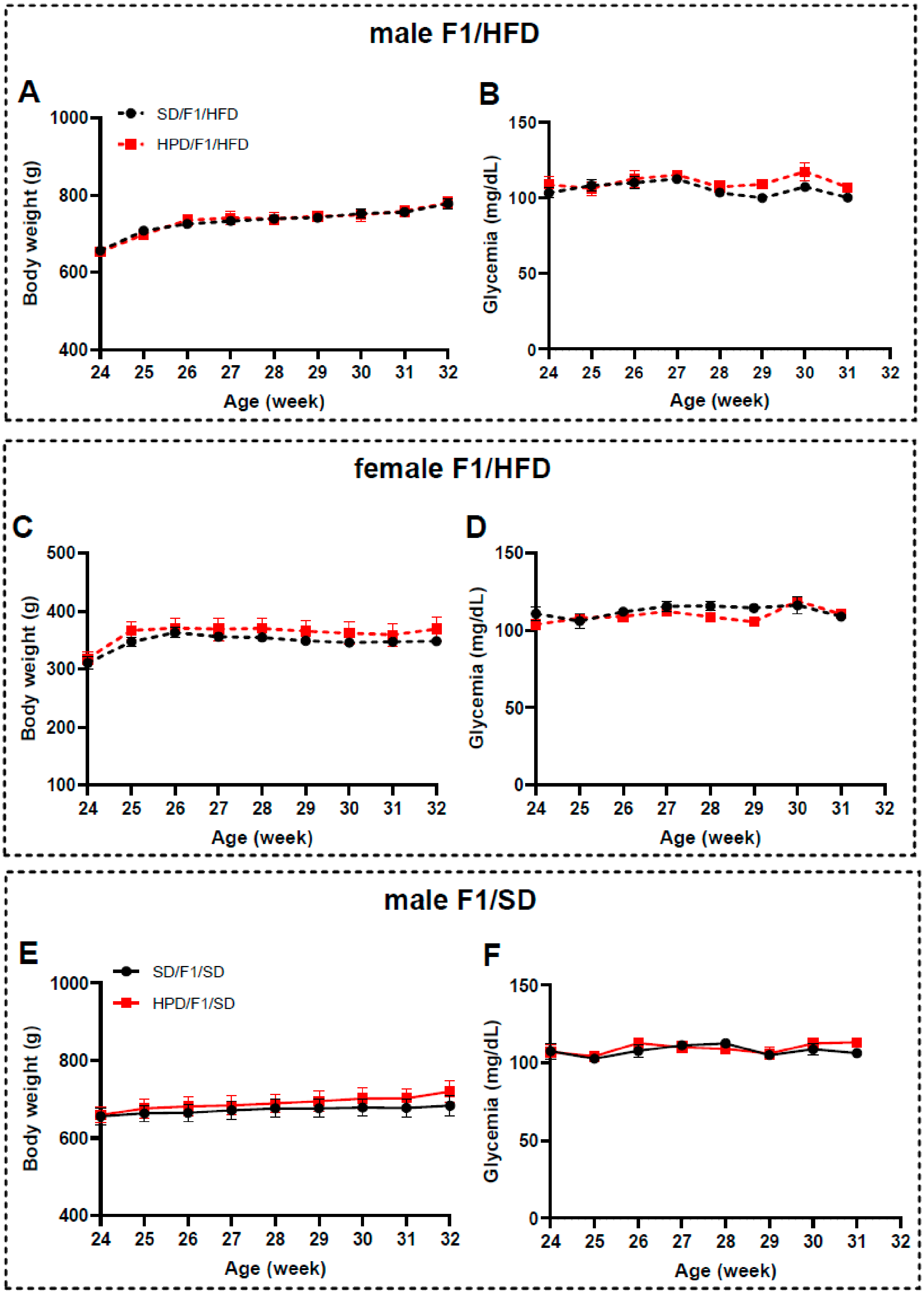
male F1/HFD
A
B
SD/F1/HFD
HPD/F1/HFD
Body weight (g)
Glycemia (mg/dL)
Age (week)
female F1/HFD
C
D
Body weight (g)
Glycemia (mg/dL)
Age (week)
male F1/SD
E
F
SD/F1/SD
HPD/F1/SD
Body weight (g)
Glycemia (mg/dL)
Age (week)

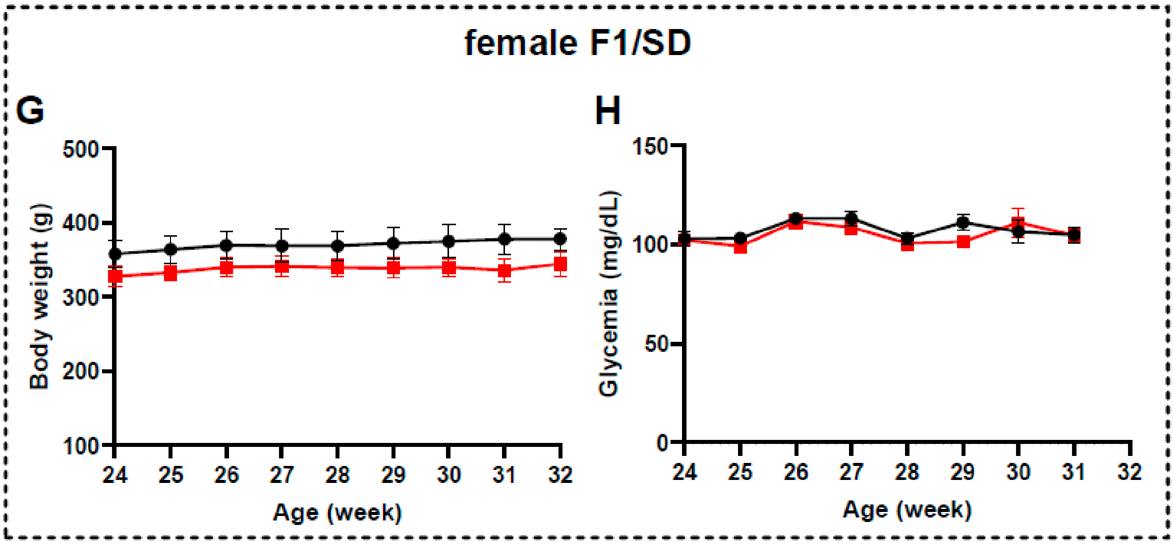

Figure S3. Evolution of body weight evolution and glycemia in male and female offspring fed with high-fat diet. Six months old male and female F1 from HPD and SD fathers were fed with high-fat diet (HFD) or standard diet (SD) for 2 months. (**A**) body weight, (**B**) glycemia of male F1 under HFD. (**C**) Body weight, (**D**) glycemia of female F1 under HFD. (**E**) Body weight, (**F**) glycemia of male F1 under SD. (**G**) Body weight, (**H**) glycemia of female F1 under SD. SD/F1/SD: offspring from SD father fed with SD from 6 to 8 months of age; HPD/F1/SD: offspring from HPD father fed with SD from 6 to 8 months of age; SD/F1/HFD: offspring from SD father fed with HFD from 6 to 8 months of age; HPD/F1/HFD: offspring from HPD father fed with HFD from 6 to 8 months of age. Four to six rats were analyzed in each experimental group. Results are expressed as means ± SEM.

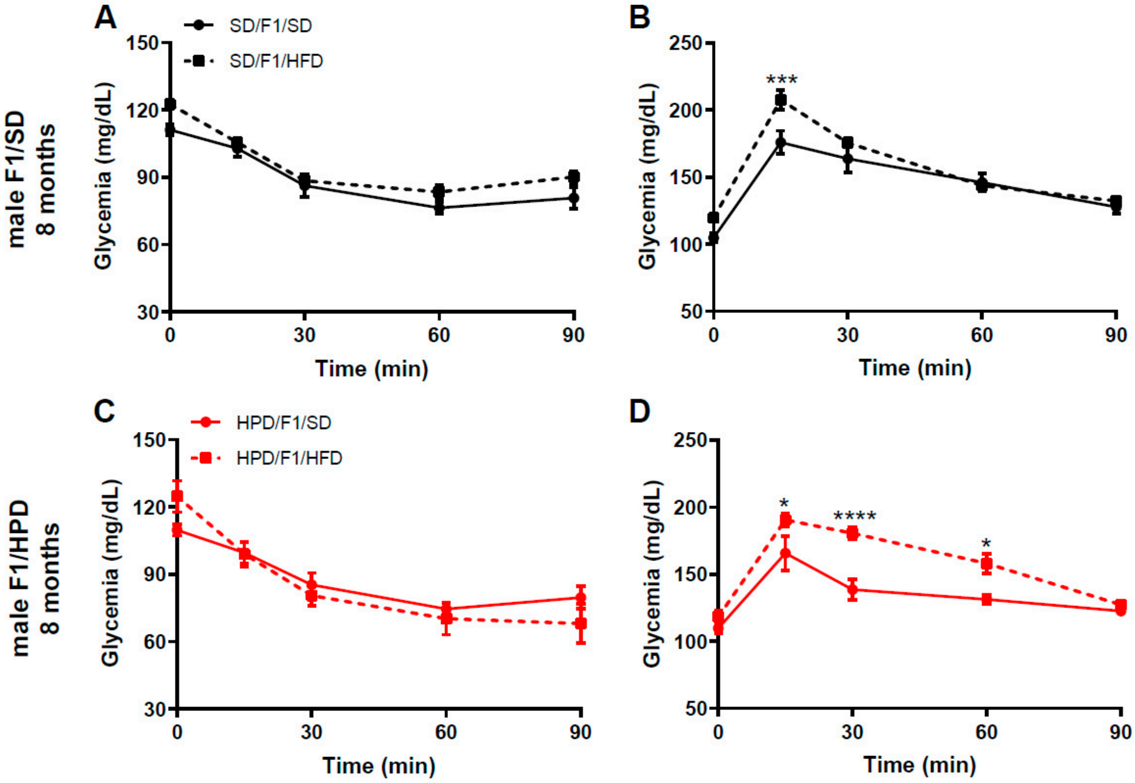

Figure S4. Insulin tolerance test and intraperitoneal glucose tolerance test in 8 months old F1 from HPD and SD fathers fed with standard diet or high-fat diet. (**A**) Insulin tolerance test (insulin, 0.5 U/kg body weight), (**B**) intraperitoneal glucose tolerance test (IPGTT, 1 g/kg body weight), in male SD/F1/SD and SD/F1/HFD, at the age of 8 months. (**C**) Insulin tolerance test (insulin, 0.5 U/kg body

weight), (**D**) intraperitoneal glucose tolerance test (IPGTT, 1 g/kg body weight), in male HPD/F1/SD and HPD/F1/HFD, at the age of 8 months. SD/F1/SD: offspring from SD father fed with SD; SD/F1HFD: offspring from SD father fed with high-fat diet; HPD/F1/SD: offspring from HPD father fed with SD. HPD/F1/HFD: offspring from HPD father fed with high-fat diet. Four to six rats were analyzed in each experimental group. Results are expressed as means ± SEM. *p<0.05, ***p<0.01 and ****p<0.0001.

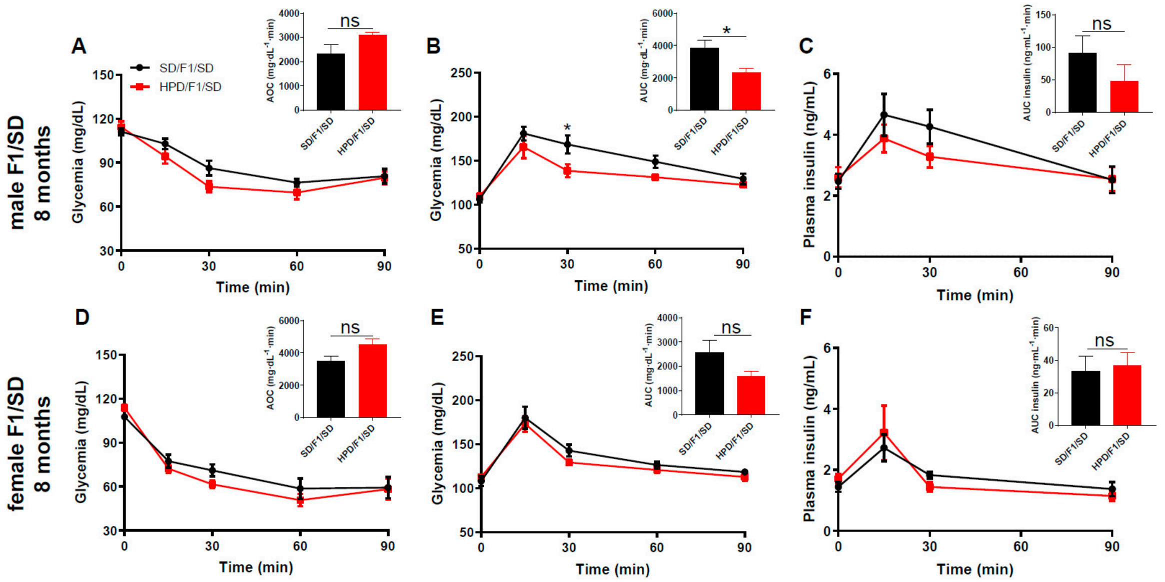

Figure S5. Parameters of glucose homeostasis in 8 months old F1 from HPD and SD fathers fed with standard diet. (**A**) Insulin tolerance test (insulin, 0.5 U/kg body weight), (**B**) intraperitoneal glucose tolerance test (IPGTT, 1 g/kg body weight), (**C**) glucose-induced insulin secretion in male HPD/F1/SD and SD/F1/SD, at the age of 8 months. (**D**) Insulin tolerance test (insulin, 0.5 U/kg body weight), (**E**) intraperitoneal glucose tolerance test (IPGTT, 1 g/kg body weight), (**F**) glucose-induced insulin secretion in female HPD/F1/SD and SD/F1/SD, at the age of 8 months. Area over curve (AOC) (A, D) or area under the curve (AUC) (B, C, E, F) are shown in the insert. SD/F1/SD: offspring from SD father fed with SD; HPD/F1/SD: offspring from HPD father fed with SD. Four to six rats were analyzed in each experimental group. Results are expressed as means ± SEM. *p<0.05.
