# Supplementary material for: Paternal High-Protein Diet Programs Offspring Insulin Sensitivity in a Sex-Specific Manner
Source: Biomolecules. 2021 May 18;11(5):751. doi: 10.3390/biom11050751 (PMC8157381; doi:10.3390/biom11050751)
Supplement: Supplementary file 1 [file biomolecules-11-00751-s001.zip › Table S2.pdf]

Table S2: Deregulated miRNA involved in insulin sensitivity. Looking in PubMed (key word "sensitivity" "insulin" "named of the miRNA), we found that 21 out 56 deregulated miRNA have been involved in insulin sensitivity (in bold).

	log2FoldChange	Padj	Reference
**rno-mir-204-5p**	**-1.007**	**0.002**	**[1]**
**rno-mir-190b-5p**	**-1.008**	**0.03**	**[2]**
**rno-mir-30b-5p**	**-1.028**	**0.003**	**[3]**
rno-mir-140-3p	-1.062	0.002	
rno-mir-451-5p	-1.066	0.003	
rno-mir-148b-3p	-1.084	0.014	
rno-mir-186-5p	-1.114	0.008	
**rno-mir-27a-3p**	**-1.188**	**0.0005**	**[4]**
**rno-mir-146a-5p**	**-1.221**	**0.0003**	**[5]**
**rno-mir-30d-5p**	**-1.265**	**0.0004**	**[5]**
**rno-mir-26a-5p**	**-1.274**	**4.13e-05**	**[6]**
rno-mir-10b-5p	-1.299	0.002	
rno-mir-181b-5p	-1.304	0.001	**[7]**
rno-mir-203a-3p	-1.332	0.004	
**rno-mir-30a-5p**	**-1.346**	**0.001**	**[8]**
rno-mir-23b-3p	-1.358	8.71e-06	
rno-mir-181a-5p	-1.378	0.002	
rno-mir-328a-3p	-1.397	0.001	
**rno-mir-24-3p**	**-1.439**	**0.001**	**[7]**
**rno-mir-27a-3p**	**-1.449**	**1.58e-05**	**[4]**
rno-mir-92a-3p	-1.461	4.26e-06	
rno-mir-10a-5p	-1.463	0.0003	
**rno-mir-24-3p**	**-1.488**	**0.002**	**[7]**
rno-mir-195-5p	-1.507	2.09e-05	
**rno-mir-361-5p**	**-1.550**	**4.36e-05**	**[9]**
rno-mir-296-5p	-1.603	0.0010	
rno-mir-23a-3p	-1.616	8.79e-08	
rno-mir-26b-5p	-1.625	7.54e-06	
**rno-mir-218a-5p**	**-1.69**	**8.03e-05**	**[10]**
rno-mir-339-5p	-1.72	2.79e-05	
rno-mir-199a-3p	-1.73	3.23e-07	
rno-mir-30c-5p	-1.74	1.81e-06	
rno-mir-196a-5p	-1.74	0.0002	
rno-mir-30c-5p	-1.75	1.72e-06	
rno-mir-196b-1	-1.77	3.50e-05	
rno-mir-196b-2	-1.77	3.50e-05	
**rno-mir-320-3p**	**-1.77**	**2.27e-05**	**[11,12]**
**rno-mir-221-3p**	**-1.82**	**1.19e-07**	**[13]**

**rno-mir-22-3p**	**-1.86**	**8.96e-06**	**[14]**
rno-mir-99a-5p	-1.92	2.788e-09	
rno-mir-222-3p	-2.006	1.03e-08	
rno-mir-100-5p	-2.048	1.19e-07	
**rno-mir-126a-3p**	**-2.050**	**6.51e-07**	**[13]**
rno-mir-223-3p	-2.070	9.44e-09	
rno-mir-99b-5p	-2.118	6.74e-11	
rno-mir-152-3p	-2.366	9.17e-09	
**rno-mir-107-3p**	**-2.679**	**3.55e-09**	**[15]**
**rno-mir-194-5p**	**-3.523**	**2.29e-13**	**[16]**
**rno-mir-378a**	**-3.566**	**4.40e-33**	**[16]**
rno-mir-194-3p	-3.953	7.82e-26	
rno-mir-455-3p	-4.021	2.09e-17	
**rno-mir-192-5p**	**-4.440**	**4.75e-43**	**[16]**
rno-mir-3068-3p	1.25	0.014	

1. Zhang Y, Gu M, Ma Y, Peng Y. LncRNA TUG1 reduces inflammation and enhances insulin sensitivity in white adipose tissue by regulating miR-204/SIRT1 axis in obesity mice. Mol Cell Biochem. 2020;475(1-2):171-83.
2. Hung TM, Ho CM, Liu YC, Lee JL, Liao YR, Wu YM, et al. Up-regulation of microRNA-190b plays a role for decreased IGF-1 that induces insulin resistance in human hepatocellular carcinoma. PLoS One. 2014;9(2):e89446.
3. Dai LL, Li SD, Ma YC, Tang JR, Lv JY, Zhang YQ, et al. MicroRNA-30b regulates insulin sensitivity by targeting SERCA2b in non-alcoholic fatty liver disease. Liver Int. 2019;39(8):1504-13.
4. Benito-Vicente A, Uribe KB, Rotllan N, Ramírez CM, Jebari-Benslaiman S, Goedeke L, et al. miR-27b Modulates Insulin Signaling in Hepatocytes by Regulating Insulin Receptor Expression. Int J Mol Sci. 2020;21(22).
5. Nunez Lopez YO, Garufi G, Pasarica M, Seyhan AA. Elevated and Correlated Expressions of miR-24, miR-30d, miR-146a, and SFRP-4 in Human Abdominal Adipose Tissue Play a Role in Adiposity and Insulin Resistance. Int J Endocrinol. 2018;2018:7351902.
6. Fu X, Dong B, Tian Y, Lefebvre P, Meng Z, Wang X, et al. MicroRNA-26a regulates insulin sensitivity and metabolism of glucose and lipids. J Clin Invest. 2015;125(6):2497-509.
7. Wang S, Liang C, Ai H, Yang M, Yi J, Liu L, et al. Hepatic miR-181b-5p Contributes to Glycogen Synthesis Through Targeting EGR1. Dig Dis Sci. 2019;64(6):1548-59.
8. Saha PK, Hamilton MP, Rajapakshe K, Putluri V, Felix JB, Masschelin P, et al. miR-30a targets gene networks that promote browning of human and mouse adipocytes. Am J Physiol Endocrinol Metab. 2020;319(4):E667-e77.
9. Zhang Z, Liu X, Xu H, Feng X, Lin Y, Huang Y, et al. Obesity-induced upregulation of miR-361-5p promotes hepatosteatosis through targeting Sirt1. Metabolism. 2018;88:31-9.
10. Lang H, Ai Z, You Z, Wan Y, Guo W, Xiao J, et al. Characterization of miR-218/322-Stxbp1 pathway in the process of insulin secretion. J Mol Endocrinol. 2015;54(1):65-73.
11. Dandare A, Rabia G, Khan MJ. In silico analysis of non-coding RNAs and putative target genes implicated in metabolic syndrome. Comput Biol Med. 2021;130:104229.

12. Beuzelin D, Kaeffer B. Exosomes and miRNA-Loaded Biomimetic Nanovehicles, a Focus on Their Potentials Preventing Type-2 Diabetes Linked to Metabolic Syndrome. Front Immunol. 2018;9:2711.
13. de Almeida-Faria J, Duque-Guimarães DE, Ong TP, Pantaleão LC, Carpenter AA, Loche E, et al. Maternal obesity during pregnancy leads to adipose tissue ER stress in mice via miR-126-mediated reduction in Lunapark. Diabetologia. 2021.
14. Thibonnier M, Esau C, Ghosh S, Wargent E, Stocker C. Metabolic and energetic benefits of microRNA-22 inhibition. BMJ Open Diabetes Res Care. 2020;8(1).
15. Yang L, Zhang B, Wang X, Liu Z, Li J, Zhang S, et al. P53/PANK1/miR-107 signalling pathway spans the gap between metabolic reprogramming and insulin resistance induced by high-fat diet. J Cell Mol Med. 2020;24(6):3611-24.
16. Latouche C, Natoli A, Reddy-Luthmoodoo M, Heywood SE, Armitage JA, Kingwell BA. MicroRNA-194 Modulates Glucose Metabolism and Its Skeletal Muscle Expression Is Reduced in Diabetes. PLoS One. 2016;11(5):e0155108.
